# Measuring Mobility and Room Occupancy in Clinical Settings: System Development and Implementation

**DOI:** 10.2196/19874

**Published:** 2020-10-27

**Authors:** Gabriele Marini, Benjamin Tag, Jorge Goncalves, Eduardo Velloso, Raja Jurdak, Daniel Capurro, Clare McCarthy, William Shearer, Vassilis Kostakos

**Affiliations:** 1 University of Melbourne Melbourne Australia; 2 Queensland University of Technology Brisbane Australia; 3 Northern Health Melbourne Australia

**Keywords:** localization, indoor, efficiency, Bluetooth, occupancy, mobility, metrics, smartphone, mobile phone

## Abstract

**Background:**

The use of location-based data in clinical settings is often limited to real-time monitoring. In this study, we aim to develop a proximity-based localization system and show how its longitudinal deployment can provide operational insights related to staff and patients' mobility and room occupancy in clinical settings. Such a streamlined data-driven approach can help in increasing the uptime of operating rooms and more broadly provide an improved understanding of facility utilization.

**Objective:**

The aim of this study is to measure the accuracy of the system and algorithmically calculate measures of mobility and occupancy.

**Methods:**

We developed a Bluetooth low energy, proximity-based localization system and deployed it in a hospital for 30 days. The system recorded the position of 75 people (17 patients and 55 staff) during this period. In addition, we collected ground-truth data and used them to validate system performance and accuracy. A number of analyses were conducted to estimate how people move in the hospital and where they spend their time.

**Results:**

Using ground-truth data, we estimated the accuracy of our system to be 96%. Using mobility trace analysis, we generated occupancy rates for different rooms in the hospital occupied by both staff and patients. We were also able to measure how much time, on average, patients spend in different rooms of the hospital. Finally, using unsupervised hierarchical clustering, we showed that the system could differentiate between staff and patients without training.

**Conclusions:**

Analysis of longitudinal, location-based data can offer rich operational insights into hospital efficiency. In particular, they allow quick and consistent assessment of new strategies and protocols and provide a quantitative way to measure their effectiveness.

## Introduction

### Background

Hospitals and clinical contexts are spaces in which physical attributes are often closely linked to organizational procedures, processes, and protocols. Thus, the movement of people within a hospital can be thought of as the physical manifestation of a particular process. For instance, when a patient visits the hospital for surgery, a particular sequence is expected to be followed: admission, preparation, anesthesia, operation, and recovery. The patient progresses along this sequence by moving through the different rooms and spaces of the hospital. Similarly, staff movement is closely linked to organizational processes. Further, these rooms are highly specialized locations and serve specific clinical and operational functions.

In this paper, we demonstrate how to take advantage of this close link between physical movement and functional and organizational processes in hospitals to generate operational insights. Specifically, we show how capturing and studying the longitudinal movement of people in a hospital can provide insights into the operational characteristics and efficiency of the hospital. We show multiple analyses where long-term mobility patterns help us quantify a hospital’s operational efficiency.

Hospitals are no strangers to localization technologies [1]. Indoor localization has seen significant technological improvements in recent years [2]—the relatively inexpensive Bluetooth low energy (BLE) beacons and Apple’s iBeacon standard have brought indoor localization closer to mainstream use. Most of the applications in hospitals and clinical settings have focused on process mining [3] or real-time localization [4], that is, locating people or assets quickly and accurately. However, the data collected by such real-time systems are typically discarded and not accumulated for long periods. One reason for the lack of interest in longitudinal analyses is that we currently lack a movement-centered representation that is flexible enough to work with a variety of localization systems, which also allows researchers to study people’s flow across indoor spaces and rooms.

Yet, outside clinical settings and health care, an increasing number of studies suggest that long-term localization data can provide meaningful insights in workplaces where efficiency is of the essence and where the flow of people and assets can be optimized by studying and analyzing their movements. For instance, construction sites aim to minimize the movement of heavy assets to reduce hazards and ensure a safer working environment, and researchers are currently working on similar projects [5]. Similarly, hospitals have an interest in reducing the downtime of operating rooms (operating rooms) as they can cost up to Aus $1500 (US $1063) per hour when in standby [6]. This means that hospitals are strongly motivated to develop policies and standards that reduce the downtime of expensive facilities such as operating rooms. However, it can be challenging to accurately describe why facilities have downtime or why patients have to wait for long periods.

### Contribution

In this paper, we present a room- and movement-centered analysis of longitudinal, indoor localization data, focusing on the semantics of hospital rooms and their role in the workplace workflow. By analyzing this data, we were able to measure the occupancy levels of different rooms, understand how much time patients and staff spent in each phase of the treatment process, and visualize and quantify the movement of patients and staff over time. We present an overview of the system we developed and deployed, the challenges we faced, and the insights we generated by analyzing the longitudinal movement data of staff and patients. We tested and validated our approach in a long-term deployment in the ward of a public teaching hospital with 7 operating theaters. Previous works have looked at gathering insights from indoor localization systems in different settings: music festivals [7], museums and galleries [8], and clinical environments [9]. However, these works do not provide a long-term analysis, focusing instead on single-time events or short-term data collection.

From a technical standpoint, the contribution of our work is in the development of algorithms that can analyze longitudinal indoor localization data and generate high-level insights with minimal training and without an understanding of the specific application domain (eg, hospitals). Researchers in the field of geographic information sciences have tackled a similar challenge of abstracting indoor spaces [10] from a theoretical point of view, which has been shown to be useful in the modeling of movements in clinical settings [11]. To the best of our knowledge, this study is the first to invert the role of Bluetooth tags and anchor nodes in a longitudinal deployment. From a clinical perspective, our work demonstrates how localization systems can be used to generate operational and efficiency measures in clinical settings.

## Methods

### Overview

We developed a system that captures the movement of staff and patients through the operating ward of a hospital, which spans a single upper floor. Our technological implementation, which we describe next, considers a range of requirements in terms of range, precision, and power consumption. During our study, the staff members we observed, including nurses, surgeons, and technical staff, had a range of roles. We also captured patients’ journeys through the various rooms of that ward, starting from the reception room to the preoperative rooms (daily process unity [DPU] for preparation) anesthetic rooms, operating rooms, and recovery rooms before discharge.

**Figure 1:**
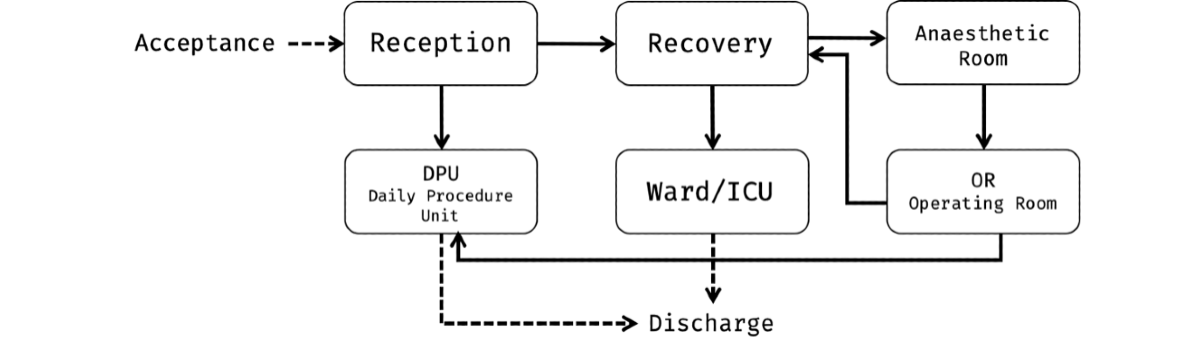
Graph representing the expected journey of a patient through the ward.

On the basis of discussions and interviews with staff members, we produced a directed graph, as presented in Figure 1, which shows the expected journey of a patient in this ward, from the moment they arrive at the reception to the time of discharge. As part of our aim to quantify the operational performance at this hospital, one of our objectives was to understand what the true patient journey looked like as opposed to the expected journey. This entailed looking at how much time each stage of the journey took and where staff members spent most of their time.

### Indoor Localization System

Hospitals are logistically challenging environments where efficiency and patient care are of utmost importance. Therefore, tracking solutions involving bulky devices that require frequent maintenance and attention are not appropriate for such a setting. Thus, the system we deployed had to be unobtrusive, effortless to manage, and reliable. Given that rooms play an important operational role in hospitals, we were only interested in capturing location at the room level and not at a more granular scale, such as the specific coordinates of the people being tracked. Therefore, we were able to use a proximity-based indoor localization system. These systems have been around for many years [12] and have been used in a multitude of scenarios without deploying substantial infrastructure [13]. The following requirements had the most significant impact on our choice of the localization system:

- Proximity-based localization systems require fixed hardware at a known location and mobile hardware that can infer its location when it comes close to fixed hardware. Due to the hospital’s hygiene policies and their privacy requirements, nurses, surgeons, and patients may not be carrying their phones at all times. Further, patients who are about to have an operation are not able to carry any devices. Therefore, in our scenario, mobile hardware (or beacon) carried by people could not be their phone, and had to be a lightweight and noninvasive device that did not distract staff and patients.
- The equipment deployed throughout the theater needed to have a battery life that kept them running and scanning the surroundings for as long as possible without reducing their accuracy.
  Because emergencies often take place, a power socket may suddenly become essential to staff members; therefore, equipment should not immediately turn off when unplugged.
  Staff members often cycle through different departments in the hospital, making it hard to inform and educate them regarding our ongoing localization project. Staff members are instructed to keep the rooms safe for patients, and any new equipment may seem suspicious or new to the staff.
  Similarly, patients might not be familiar with such devices, as they might unplug the anchor nodes or turn off their tracking device.
- We wanted to minimize the deployment of infrastructure, which can be disruptive and costly. A number of localization systems rely on Wi-Fi infrastructure, which is already present. However, this requires people to carry their own mobile devices, which is not always possible or desirable. Similarly, radio-frequency identification (RFID) systems have been used for localization, but they tend to have either a very short range of a few centimeters (passive RFID) or be heavy and noisy (active RFID) [14].

**Figure 2:**
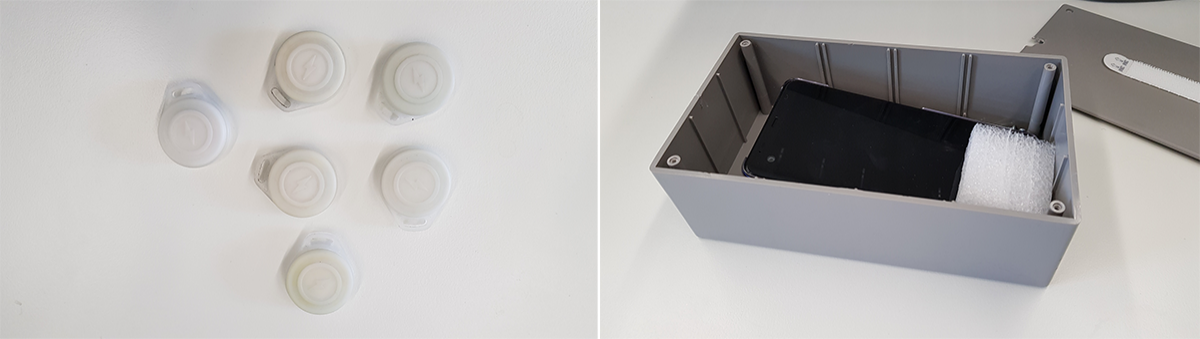
Beacons handed out to staff and patients; android devices that get mounted to the walls.

Considering the above requirements in terms of range, precision, and power consumption, we developed a proximity-based localization system that uses BLE [15]. The system consists of Android smartphones deployed as fixed anchor nodes throughout the hospital floor and RadBeacon Dot iBeacons [16] handed out to patients and staff members, as shown in Figure 2. The beacons are small and light enough to be attached to the staff badge or patient bracelet. This potentially increases adherence rates, as badges and bracelets are mandated to be worn at all times.

Typically, BLE-based localization systems rely on beacons as fixed anchor nodes and smartphones as carried devices [17]. Due to the constraints we have identified, we opted for an inverted set up where staff members and patients carry a BLE beacon, while smartphones act as fixed anchors strategically placed throughout the hospital. To the best of our knowledge, this is an approach that has not been tested before in a longitudinal study.

### Software

**Figure 3:**
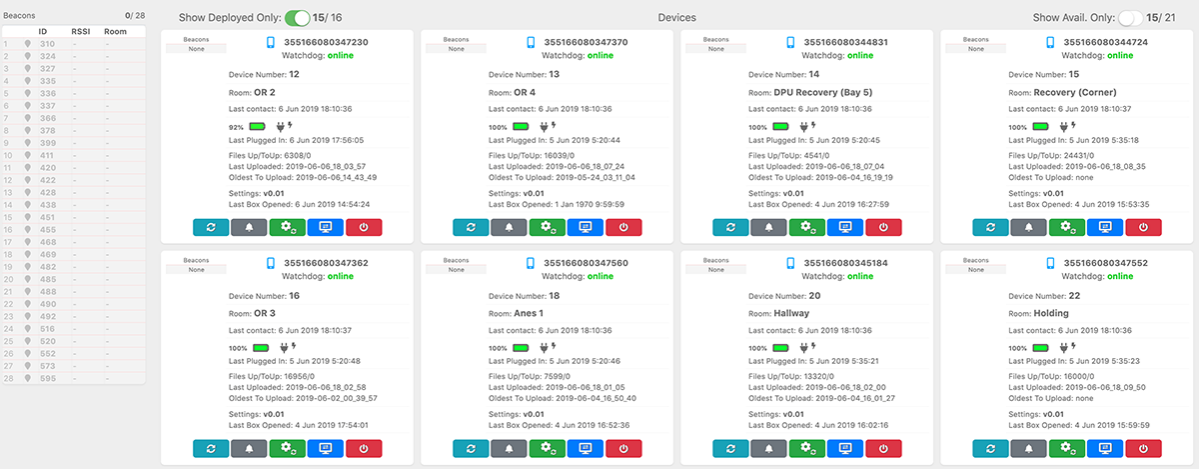
Snapshot of the web-based dashboard used to monitor the deployment and data collection. This was used to ensure the deployment runs as expected.

We developed a custom Android app that continuously runs a low energy scan to detect all nearby beacons. The app continuously runs on the phone and locally stores the received signal strength indication (RSSI) readings of the received Bluetooth packets, along with the timestamp in epoch milliseconds, the minor identification number of the iBeacon emitting the packet, and the International Mobile Equipment Identity of the smartphone itself. On a daily basis, each smartphone uploaded a compressed data file containing the collected data to our server. A web-based dashboard allowed us to monitor our deployment, check the state of the smartphone network, and check for irregularities or mistakes in the smartphone and beacon labeling and configuration. Smartphones sent a brief update on their own state, including the last group of beacons they scanned, their battery level, and charging state, to the dashboard every few seconds. A screenshot of the dashboard is shown in Figure 3.

### Beacon Configuration

According to the manufacturer’s specifications, the beacon batteries can last between a month and more than a year, depending on the signal power and the advertisement rate to which the beacon is set. A lower power or advertisement rate allows for longer battery life, but the beacon emits its identity less frequently, meaning that in cases where a beacon is moving very fast, the anchor point can miss it. Conversely, a higher power and advertisement rate improves the chances of a beacon being detected at the cost of fairly short battery life.

**Figure 4:**
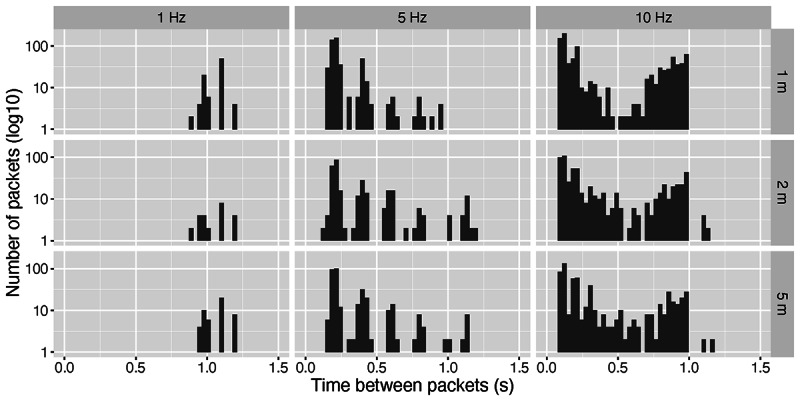
Laboratory measurements for different beacon advertisement rates (1 Hz, 5 Hz, and 10 Hz) and distance between the beacon and anchor node (1m, 2m, and 5m). We identified 5 Hz as the optimum setting, in terms of performance and battery consumption.

Due to the way the hardware is built, the advertisement rate needs to be set before the experiment. For this reason, we conducted laboratory measurements to identify the optimal beacon settings for our scenario. We assumed beacons to be moving at walking speed and wanted them to be detectable at room-size distances, in our case, up to 5 m away. We configured beacons at different advertisement rates (1 Hz, 5 Hz, and 10 Hz) and placed them at varying distances from an anchor node (1 m, 2 m, and 5 m). In Figure 4, we show the effect of these 2 variables (advertisement rate and distance) on how the anchor node can detect those beacons (ie, the time between consecutive received packets). Effectively, this is a measurement of the number of packets lost. Given these experimental conditions, we identified that the optimum setting for our scenario, in terms of battery consumption and packet loss, is 5 Hz. At a frequency of 1 Hz, we observed an undesirable loss of packets; at a frequency of 10 Hz, we observed no noticeable performance improvement, whereas the drain on the battery doubled.

### Deployment

**Figure 5:**
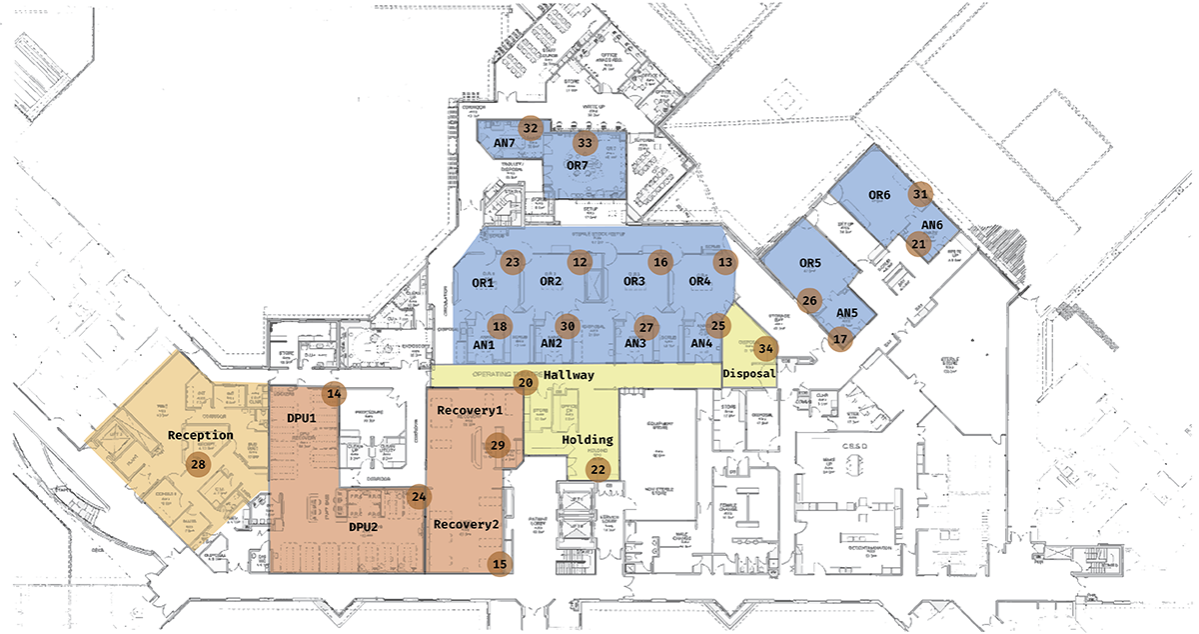
Unfiltered received signal strength indication readings and ground truth (gray line) for a single beacon moving across rooms. X-axis: time; y-axis: room identifier; color: RSSI strength (blue: weak; red: strong). RSSI: received signal strength indication.

The project was approved by the hospital’s Health Human Research Ethics Committee. Each fixed anchor node comprises a plastic container, as shown in Figure 2 containing an Android HTC U11 smartphone. We deployed our anchor nodes throughout the ward (Figure 5) and plugged them into the nearest wall socket. Because our objective was to detect the presence of a beacon inside any given room, we placed 1 box in each room of interest after confirming with the staff that the box would not cause any inconvenience to them or the patients and that a nearby power source was available. In most of the rooms, the box was placed under a desk or mounted on a wall.

**Figure 6:**
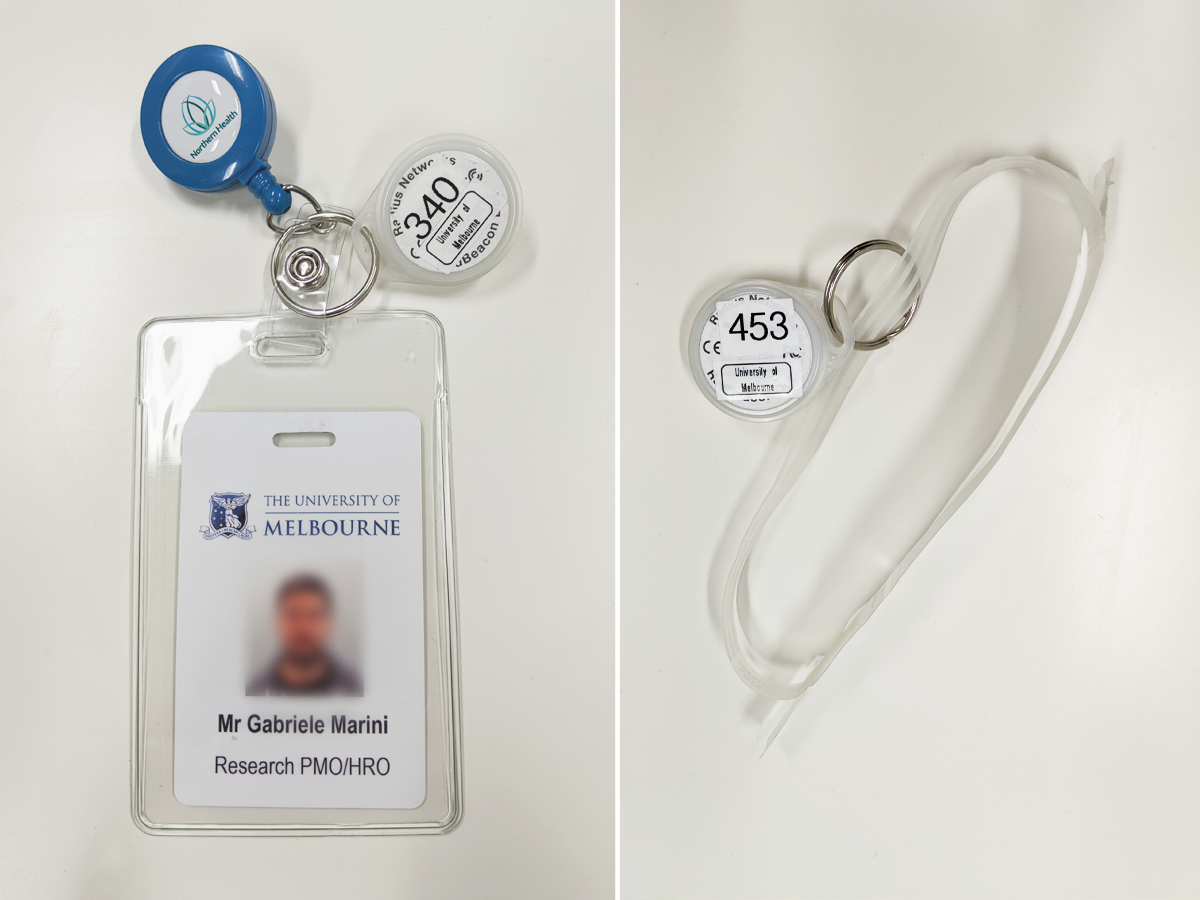
Examples of how beacons were handed out. Strapped to a staff badge or to a patient's bracelet.

We handed out RadBeacon iBeacons to staff members (nurses, theater technicians, and head theater technicians) who attached them to their badges, as shown in Figure 6. Staff members are mandated to carry their badges all the time, usually on their front pocket, or sometimes in their bottom or trouser pocket. When a beacon was handed out, we made a manual spreadsheet entry to link the beacon ID to the staff ID. This allowed us to link the beacon ID to staff roles during our analysis. Staff was instructed to keep the same beacon during the study and make a new data entry if they were issued a new beacon.

Patients received their beacon along with their patient bracelets (Figure 6), as part of the hospital standard admission procedure. To identify patients, the hospital uses plastic bracelets, which strapped to their wrists during admission and cut off when they are discharged. When nurses gave a beacon to a patient, a spreadsheet entry was manually made to link the beacon ID to the patient ID. This allowed us to identify which beacon belonged to which patient during our analysis.

### Ground-Truth Collection

Before we began our main deployment, we wanted to validate the data generated by the system. Ground-truth in localization refers to the true coordinates of an entity being localized. Ground-truth data may be collected by researchers independently through reliable and verifiable means, and is used to validate the correctness of the localization algorithm and fine tune its parameters. We systematically collected ground-truth data in multiple sessions. A researcher carried beacons with them and traversed the space while manually logging their precise location and exact time using a smartphone app. In total, we collected ground-truth data from 18 sessions, each lasting about 15 min and collected on a single day. All sessions were quite dynamic, meaning that the researcher moved continuously between rooms, rather than remaining static at a single location. During these sessions, we simulated realistic scenarios such as walking down the halls, entering an operating room, roaming around the surgery table, and eventually leaving and getting out of the ward and the tracked area.

Using this ground-truth data, we were able to use our system and test various filtering and analysis techniques until the trips captured by the system accurately reflected the ground truth. We present our results in the next section.

## Results

Our main deployment lasted for 30 days in September 2019. The deployment consisted of 20 anchor nodes installed in various rooms of the operating ward, including 7 operating rooms. We collected a total of 35 million packets emitted from 66 beacons handed out to 75 different people during this period. Some beacons were reused, whereas others were replaced. They were given to 17 patients by reception nurses (who also retrieved them when a patient was discharged), 15 nurses, and 7 theater tech staff.

### Collected Raw Data

**Figure 7:**
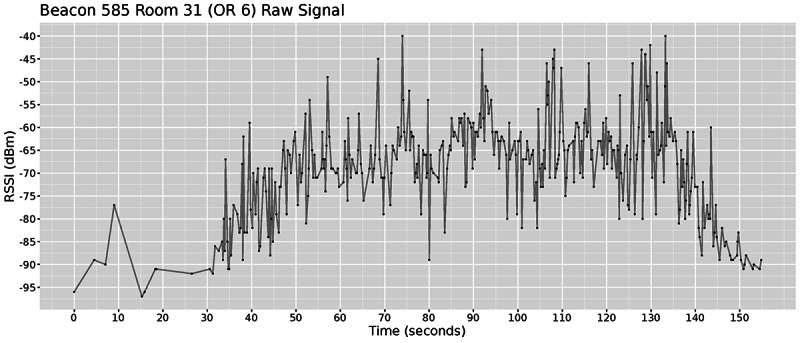
Received signal strength indication for a single beacon in a single room for a period of 3 min. A stronger signal suggests that the beacon was closer to the anchor node. RSSI: received signal strength indication; OR: operating room.

At the most basic level, our system captures raw signal strength data, also known as RSSI. For example, Figure 7 shows the RSSI for beacon 585 in the operating room.

There is substantial literature on how to use Bluetooth for indoor localization purposes, and the reported applications range from a simple but less accurate triangulation [18] to more accurate but laborious finger-printing [19]. For our purposes, we were interested in making room-level inferences about individuals’ presence because rooms are strongly linked to organizational processes. This means that we did not need to use triangulation (which requires the deployment of multiple anchor nodes), but could rely on single proximity measurements to infer the room within which an individual is. Bluetooth measurements are notoriously prone to unreliable RSSI readings [20] because of the physical nature of electromagnetic signals. The measurements can be affected by the mere presence of a human body or furniture, and especially walls and floors can attenuate the received signal thus altering the final RSSI reading. We actually used these limitations to our advantage by effectively trapping the signal within our areas of interest, and took advantage of the fact that signals traveling through walls are substantially reduced in strength.

**Figure 8:**
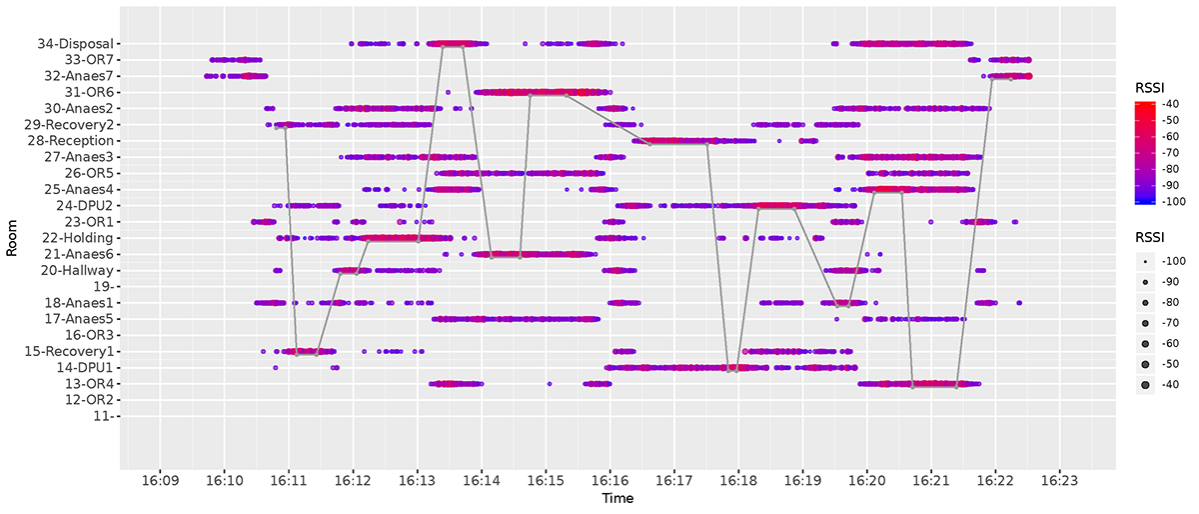
Unfiltered RSSI readings and ground truth (grey line) for a single beacon moving across rooms. X-axis: time; y-axis: room identifier; colour: RSSI strength (blue: weak; red: strong).

Although walls and floors work as natural filters, a post hoc filter is still necessary because open doors or larger rooms can cause the signal to bounce off the walls and end up in an adjacent room. We visualize this phenomenon in Figure 8, where we show the raw data collected by all anchor nodes in a span of 13 min for beacon 585. In this graph, we show along the y-axis the room identifier where beacon 585 was actually detected. The colored circles indicate the detection and strength of the signal, with purple showing a weak RSSI and red indicating a strong RSSI. The graph shows a gray line corresponding to the true path (ground truth) that the beacon took through space during this 13 min. As the beacon moved between rooms (gray line), multiple anchor nodes were able to detect that beacon (colored circles). The graph also shows that while the beacon is in a particular room, the signal strength for the anchor node in that room is higher (bright red circles). Given the amount of noise in this data, we needed to apply filters to estimate the path of the beacon through the various rooms of the hospital.

### Localization Accuracy

**Figure 9:**
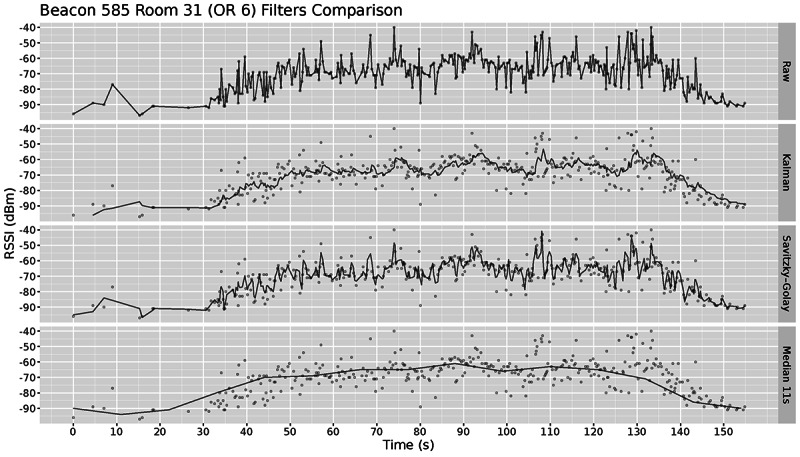
Comparison of three approaches to filter received signal strength indication data. RSSI: received signal strength indication; OR: operating room.

Bluetooth-based positioning often uses Kalman filters to smooth the RSSI values and remove outlier readings. Previous work [21] shows that although it can improve accuracy, in certain cases, a median filter can work just as well, but it does not modify the data. The purpose of filtering is to remove noise so that the location of a person in a room can be confirmed at the correct time and with significant signal strength. We evaluated 3 different filters and compared their results with our collected ground-truth data. Figure 9 shows how the different filters smoothed the signal for the data presented in Figure 7. The Kalman and Savitzky–Golay filters (second and third row) were applied using their respective R package, whereas the median filter (fourth row) was manually implemented to maximize flexibility. As expected, the results in Figure 9 show that the Kalman and the Savitzky–Golay filters fit the data correctly but fail to smooth out the signal fluctuations enough to provide a clear pattern, whereas the median filter provides a smooth estimate that makes it more appropriate for comparison. In our case, we desired smoothed values because if the data from different rooms fluctuate substantially, then we were likely to have interference and infer that the beacon was moving back and forth between the 2 rooms.

**Figure 10:**
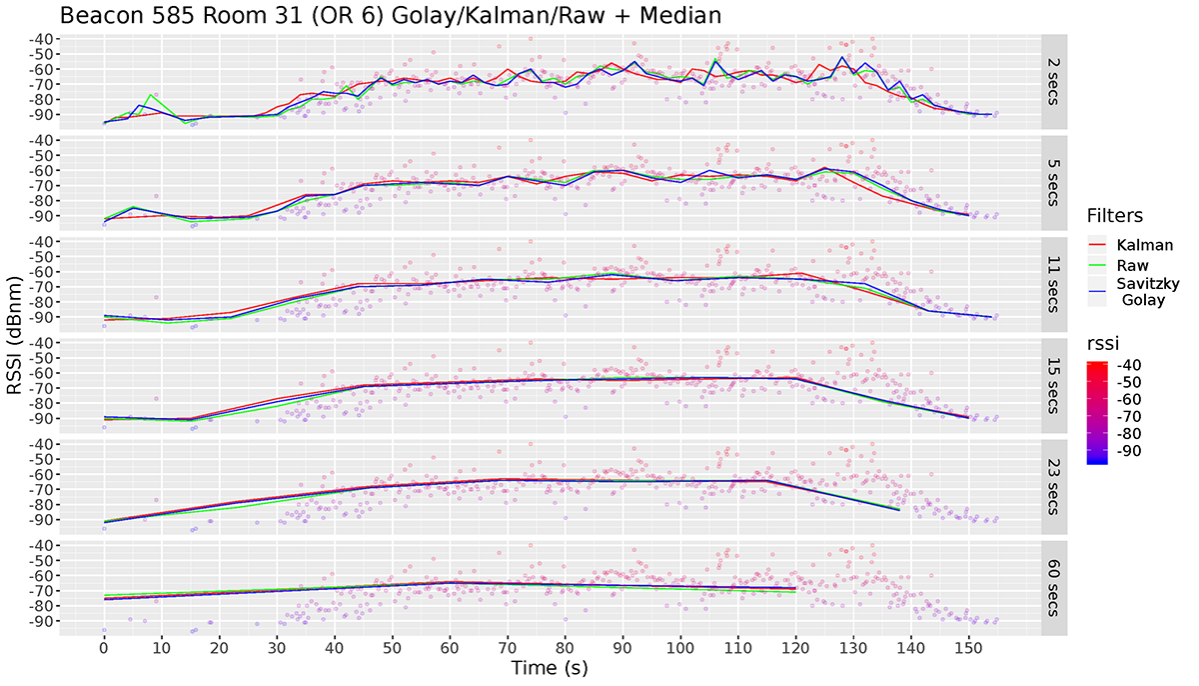
Comparison of using only the median filter (green line) versus using it in conjunction with other filters (red and blue). We test performance at different window sizes. RSSI: received signal strength indication.

Next, we needed to determine (1) whether we should use median filtering on its own or combine it with another filter and (2) what time window our median filter should use. These decisions were made based on the analysis shown in Figure 10, where we show the performance of the median filter when applied to the raw data and prefiltered data. We also investigated the effect of different window sizes (between 2 and 60 seconds). Our results show that the addition of a Kalman or Savitzky–Golay to the median filter did not improve the accuracy. This means that our median filter was robust. We also observed that when using a smaller median time window, the resulting readings fluctuated considerably, which may lead to interference across adjacent rooms. On the other hand, a larger median time window could filter out most of the noise and short bursts of high RSSI packets, but in turn, it also lost the ability to detect granular movement; a 60-second window will inevitably miss smaller events of 10 or 20 seconds. For instance, a staff member visiting a particular room for 20 seconds, and then moving to another room may not be detected using a 60-second filtering window.

We used our ground-truth data to evaluate the accuracy of our system using a range of different window sizes. There is no universally accepted approach to measuring the accuracy of data such as ours. Therefore, we adopted an approach in which we penalized our system for inferring the wrong location at any given time, but we did not consider the magnitude of the error. To measure the system accuracy, we split the time series into intervals of 1 second and compared the ground-truth data with the reconstructed trace using a string comparison algorithm (Jaro–Winkler with a prefix scale of 0.25 and cosine distances) [22].

We observed that the optimum filtering window size was 15 seconds with the median filter, giving an accuracy of 96%. For smaller window sizes, the accuracy drops by up to 10%. We, therefore, used this window size in all our subsequent analyses, meaning that visits to a room that lasted less than 15 seconds may not be captured, but we will have stronger confidence in the visits that do indeed get captured.

### Visualization of Movement

**Figure 11:**
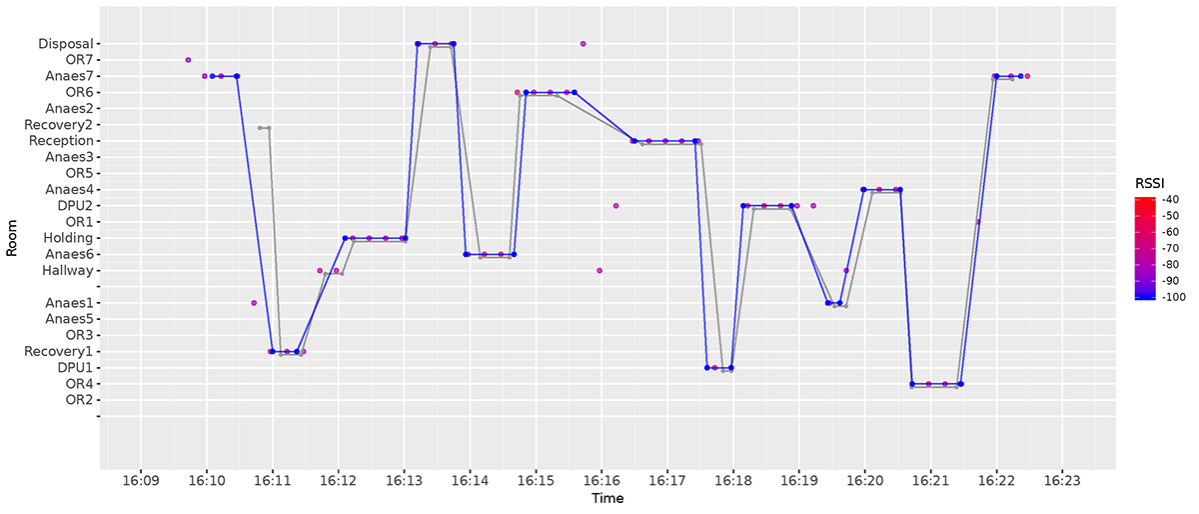
Trace reconstruction from our data, as compared to ground truth (grey line).

Figure 11 shows how our trace reconstruction process was compared with the ground truth. This figure uses the same dataset that we have used in all previous figures so far. We note that this graph shows data for 12 min during which time the position of the beacon was inferred to be in the wrong room for about 30 seconds. We also observe that our estimate sometimes appears to be slightly offset from the ground truth, suggesting that transitions between rooms were shifted by a few seconds rather than misclassified, meaning that our approach would not substantially affect the longitudinal analysis.

**Figure 12:**
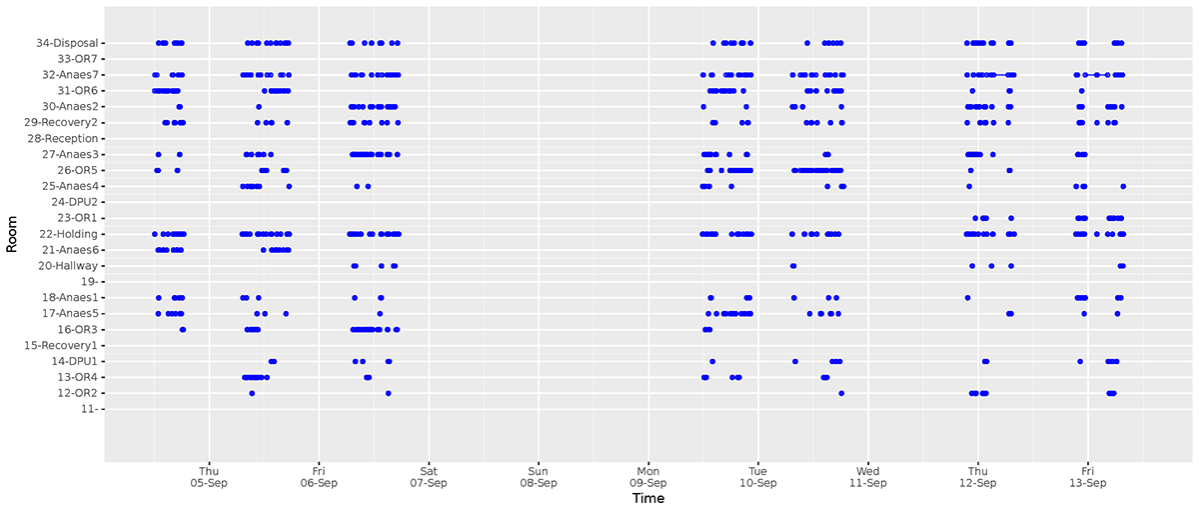
A reconstructed trace for a single nurse over a 10-day period. X-axis: date/time; y-axis: room.

Using this trace reconstruction approach, we then turned to visualize and analyze data for longer periods. In Figure 12, we show the movement of a single nurse during a period of 10 days. From this graph, we can make a number of inferences. First, we can see that the nurse typically was at the operating ward from 9 am to 5 pm, and we can also identify the days when she was not there (eg, weekends). We can also see that she appears to spend more time in certain rooms (eg, 22-holding), whereas she rarely visited other rooms (eg, 12-OR2). We can also see differences in the patterns between days. For instance, on Monday and Tuesday, the nurse spent a lot of time in room OR5 (operating theater 5).

This graph demonstrates 2 things. First, it highlights the richness in the collected data and indicates the types of inferences that can be made from the data’s temporal and spatial dimensions. Second, it demonstrates how visualizing movement traces does not scale, and indeed it becomes impractical to attempt to visualize all collected data during the whole month. Therefore, we must develop ways to summarize all our reconstructed trips and find meaningful ways to interpret data over long periods.

### Operational Insights

**Figure 13:**
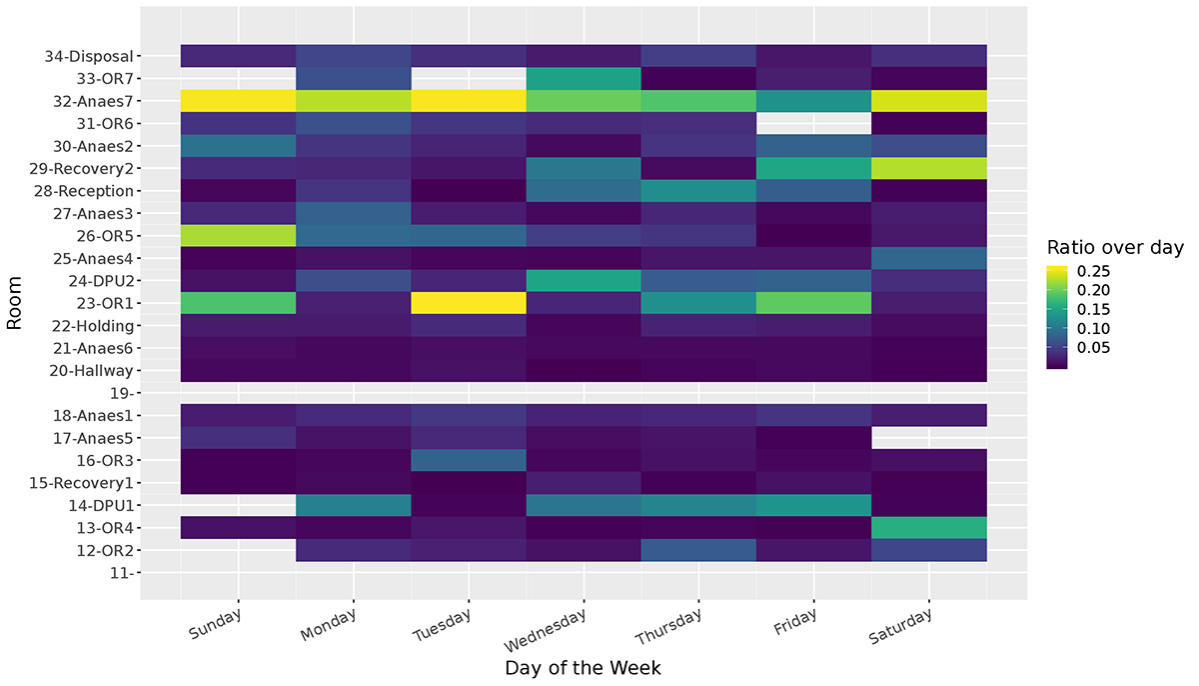
Room occupancy of nurses over a week.

**Figure 14:**
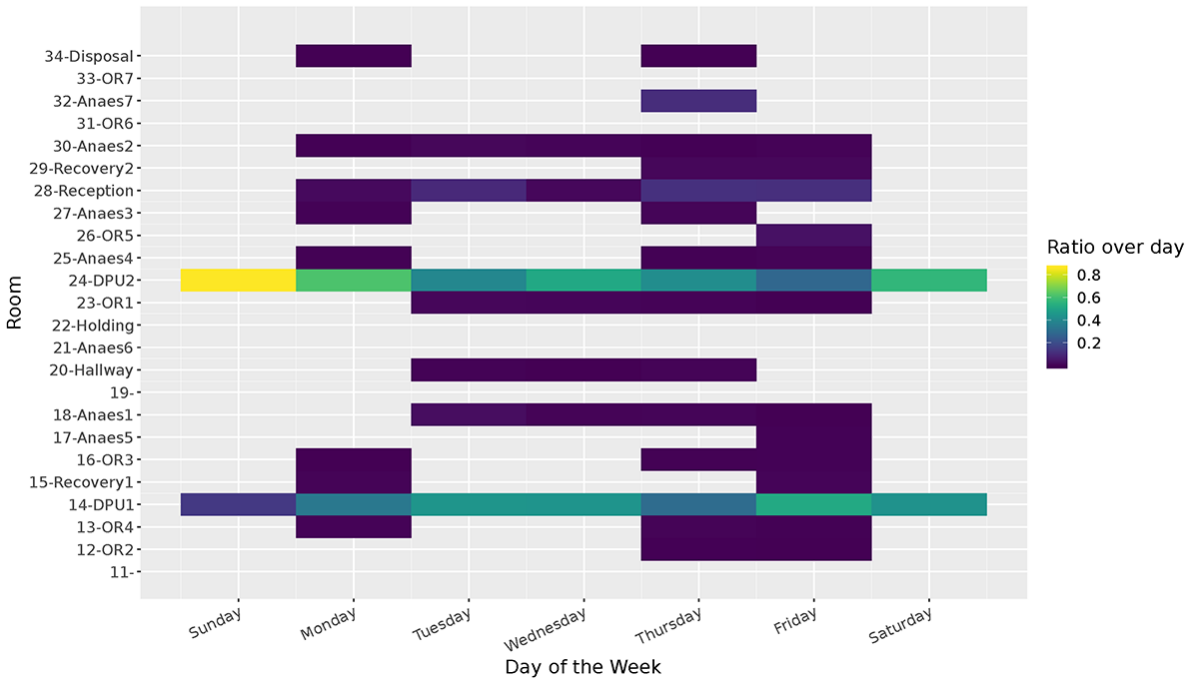
Room occupancy of patients over a week.

Representing the data as a time series of room-based events makes it easy to visualize patterns in the way people move through the spaces. Figure 12 shows the reconstructed traces of a single nurse for 10 days. In this figure, we can identify the daily and weekly patterns of the nurse and identify the rooms where most of the time was spent. We repeated this process, and in our analysis, we reconstructed all trips for all beacons and people across the entire duration of the study. Due to the richness of the data, it becomes inconvenient to visualize the data from all participants in this manner. Therefore, we sought alternative visualizations that could more clearly highlight patterns for the entire dataset. We achieved this by visualizing the occupancy rates of different rooms by different types of people. In Figures 13 and 14, we visualize the amount of time spent in each room by nurses and patients. This visualization confirms that there is a clear distinction between nurses and patients and the way they share their time across rooms during the week.

All patients in this study were daily cases and therefore spent most of their time in the DPU room. Relatively speaking, only a small fraction of their time was spent in the operating room or the anesthetic room. In addition, this visualization indicates that some operating rooms were used more than others. For instance, OR1 was often used multiple times in the lapse of a week, whereas the same cannot be said of OR6.

It is important to highlight that our deployment did not recruit 100% of staff and patients. This means that it can be misleading to calculate the absolute occupancy rates of rooms. For this reason, in Table 1, we show how much time in total was spent in a given room by different types of people during our deployment. A person-day consisted of 86,400 seconds (ie, 24 hours) of presence by a single person. This is a more meaningful metric that can be used to compare across rooms. Here, we see that OR1 had the highest occupancy by staff, and the DPUs had the highest occupancy levels during our deployment. In addition, we see how much time, on average, patients spent in different rooms.

**Table 1:** Occupancy of different rooms, measured in person-days. Overall occupancy was made up of staff occupancy plus patient occupancy levels.

Room	Overall occupancy	Staff occupancy	Patient occupancy
28, reception	0.93	0.49	0.44
18, AR^a^1	0.53	0.47	0.06
30, AR2	0.57	0.54	0.03
27, AR3	0.42	0.40	0.02
25, AR4	0.23	0.22	0.01
17, AR5	0.23	0.19	0.03
21, AR6	0.09	0.09	0
23, OR^b^1	1.39	1.37	0.02
12, OR2	0.34	0.34	0
16, OR3	0.26	0.25	0.01
13, OR4	0.16	0.14	0.02
26, OR5	0.83	0.81	0.01
31, OR6	0.87	0.87	0
33, OR7	0.45	0.45	0
14, DPU^c^1	2.94	0.60	2.33
24, DPU2	3.43	0.65	2.78
34, disposal	0.46	0.46	0
22, holding	0.45	0.45	0
15, recovery 2	0.16	0.15	0.01
29, recovery 1	0.65	0.61	0.04
20, hallway	0.11	0.10	0.01

^a^AR: anesthetic room.

^b^OR: operating room.

^c^DPU: daily process unity.

From our analysis, we could calculate how much time, on average, patients spent in different rooms of the hospital, which is indicative of how long each stage of the process took. For instance, we find that patients spent an average of 42 min (SD 55 min) at reception, 8 to 50 min in an anesthetic room (SD 5-28 min), 5 to 20 min in an operating room (SD 2-8 min), 6 to 9 min in recovery (SD 2-8 min), and 3 to 4 hours (SD 14-22 hours) in the DPU before they were discharged.

**Figure 15:**
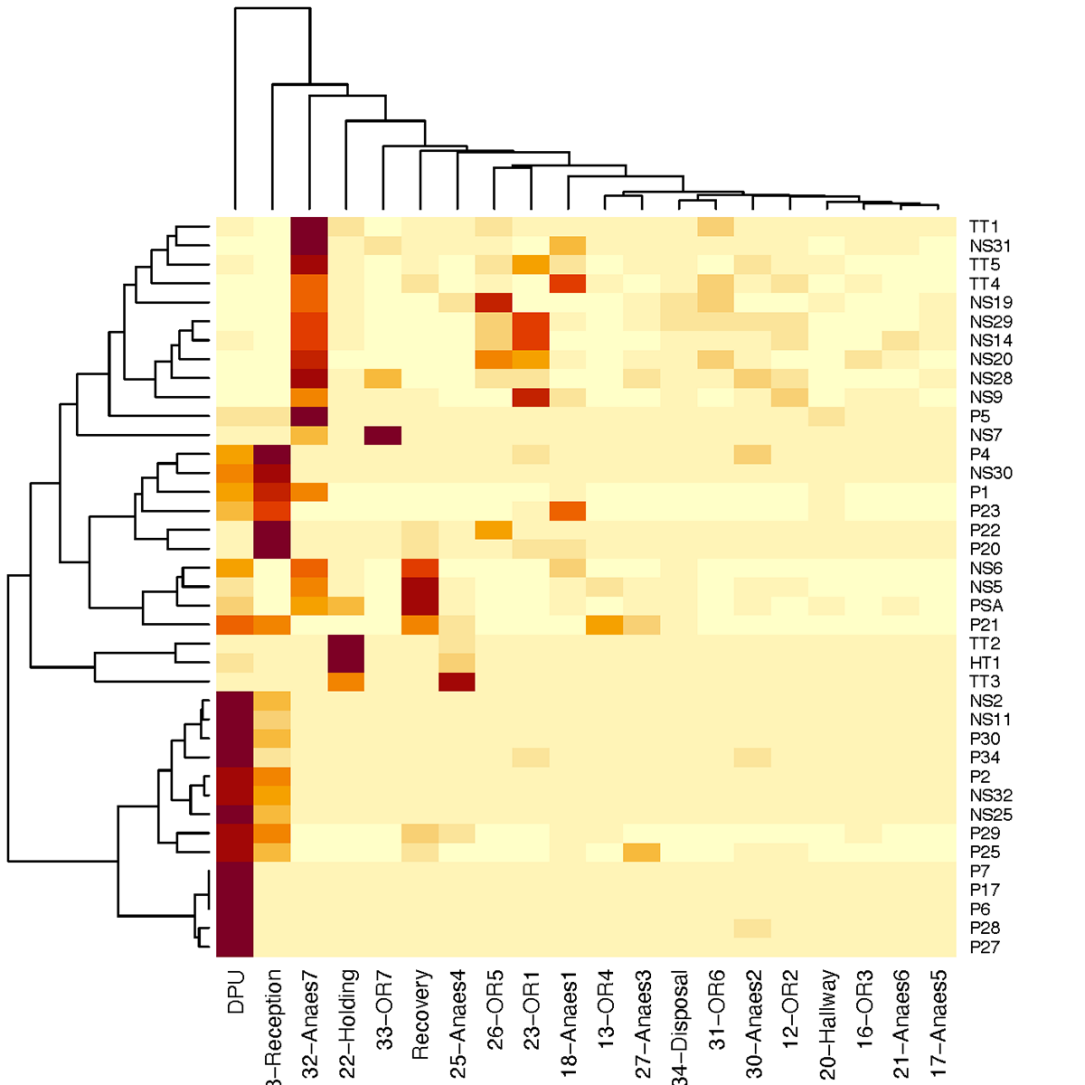
Using hierarchical clustering to group people and rooms into similar clusters. The data used for clustering is the time spent per room by each person tracked. NSxx: medical staff; Pxx: patients; TTxx/HTxx: technical staff.

Finally, our results show that each person may spend a different amount of time in different rooms. Effectively, the variations to these patterns can be thought of as a *signature* of the person’s role and function. To demonstrate this, we applied a very simplistic, unsupervised clustering to the data from Figures 13 and 14, and the results are shown in Figure 15. We fed the clustering algorithm an array of occupancy rates that describe how each person spent their time across different rooms. The algorithm then clusters people based on these values (shown as the dendrogram on the y-axis). We found that the algorithm, without any additional input or training from us, could identify 2 clusters of patients and 2 clusters of staff (medical staff and technical support staff).

## Discussion

### Indoor Localization in Clinical Settings

The use of indoor localization technologies is not new to clinical settings. In the past, most of the applications in hospitals and clinical settings focused on real-time localization. In fact, there are multiple commercial systems or indoor localization technologies in clinical settings. For instance, IBM developed the Real-Time Asset Locator

System that relies on ultrasound provides high spatial accuracy at a substantial infrastructure investment [23]; a similar system was developed by CenTrak using Bluetooth to provide real-time localization services [24]. In addition, a number of research papers have investigated the use of Bluetooth for real-time localization [25], while also focusing on particular spaces such as operating rooms [26] or particular patients, such as newborns [27].

The popularity of real-time localization systems is increasing because they can provide a number of valuable capabilities. For instance, they allow staff to quickly call for help, raise alerts when a patient has wandered into an inappropriate room, and quickly find mobile equipment or assets when needed. However, these systems do not typically provide an aggregated overview and analysis of operational measures and do not provide enough information to make grounded judgments about the processes being followed in the hospital. For this reason, hospitals typically undergo short, intense bursts of observations to map their activities and movements. These typically last a couple of weeks and rely on manual data collection, which can be costly, time-consuming, and subject to the observer effect. In addition, some systems such as TimeCaT [28] do allow staff to indicate their location and activity, but this requires ongoing manual data entry, which can be time-consuming for staff.

Manual observation stints remain popular because longitudinal data are useful for measuring and improving efficiency. In fact, in other process-based disciplines, efficiency has been improved using longitudinal analyses, including public transport [29], traffic routing [30], and construction [5]. Therefore, our paper’s premise is that analyzing long-term mobility patterns can help us quantify a hospital’s operational efficiency in multiple ways.

### System Performance

System accuracy was assessed using our ground-truth data before the main deployment. During the deployment, we considered the reliability and robustness of the system. In general, we found that the system accuracy was consistently high across all ground-truth tests (96%) in terms of room-level localization [31]. During the actual deployment, there were a number of challenges faced. For example, some staff lost or misplaced their beacon and had to be issued another one. In these cases, we noted the time when the old beacon was lost and when the new beacon was issued, and these 2 data points allowed us to seamlessly analyze the movement of any staff member. However, these incidents point to a weakness of the system and indoor localization in general [32], in that it does not work for people who do not carry their tags. We also found that our algorithm seems to identify trips that are temporally shifted by a few seconds from the ground truth. This is a side effect of using a temporal window to apply the median filter [33,34], and we believe that in terms of longitudinal analysis it does not significantly affect the findings. We also found that the sampling rate of the beacons was adequate, and the beacons did not face any battery or power issues during our deployment, as expected [35].

### Operational Insights

Our study demonstrates how longitudinal data from a proximity-based localization system can be filtered, aggregated, and analyzed to estimate relevant operational metrics related to mobility and occupancy rates. Specifically, we showed that the system could estimate how much time, on average, patients spent at each stage of their treatment process, which rooms they spent the most time in, and which stage seemed to take most time. With a larger sample, it would be possible to break down these results by particular conditions (eg, heart surgery and hip surgery) and identify trends or outliers within those.

Our system could also provide the same metrics for staff, and we could estimate where the staff spent their time and identify daily and weekly patterns in their behavior. We showed that different types of staff (medical vs technical support) exhibited different movement patterns, and in fact, a simple hierarchical clustering was able to identify the presence of these 2 staff groups.

These metrics can be used to assess the impact of a new strategy or protocol in the hospital. For instance, the metrics can be compared before and after a new scheduling system is deployed at the hospital. The comparison could be used to judge whether patient journeys are affected (eg, they spend less time at reception) and whether staff working patterns have substantially shifted (eg, staff spends relatively more of their time in the operating theater). In addition, it is also possible to characterize and study the behavior of other relevant subgroups of staff, such as junior medical staff. Their behavior can be aggregated and analyzed to identify how and where they spend their time, and to ensure that their time is spent effectively and with adequate support.

### Localization Technologies and Representations

From a localization standpoint, our work does not break new ground in terms of accurately determining the location of people. Perhaps one unique aspect of our work is our decision to use an *inverted* deployment, whereby Bluetooth tags were given to people, and phones were glued to the wall. Typically, the opposite occurs; for example, in music festivals [7] or museums and galleries [8], Bluetooth tags are installed near items of interest while people carry phones that display information for nearby items. Our decision meant that there was minimal disruption to staff and patients, who had to simply carry a light Bluetooth tag.

However, a technical contribution of our work is our development of a movement-centered representation that is flexible enough to work with a variety of localization systems and settings. This allows our system to be easily redeployed to other settings, such as schools, universities, and galleries, with minimal changes. This is possible because we have developed a data representation that allows us to calculate all the measures we have presented in this paper, but which remains agnostic of the space and environment where the deployment takes place. Effectively, this representation allows researchers to study people’s flow across abstracted rooms and spaces. Our work bears a resemblance to temporal abstraction rules [10], which can be applied to a variety of environments [36], for example, previous research [11] on a knowledge-based temporal abstraction of clinical phenomena. However, this approach relies on time-stamped clinical data, which are, in part, electronic patient records. Similarly, work on business process management [37] relies on electronic records to model corporate operations and functions, and recently, this work has been applied to clinical settings [38].

Our work, on the other hand, could capture information that was not necessarily part of electronic patient records, and which would be too costly to acquire manually. By capturing movement, we could make inferences about patient journeys and staff work practices. Similar work has recently looked at using Bluetooth to assess the levels of physical activity of people moving inside buildings and consider abstract spaces in graph form [9]. That work was confined to a handful of small trials, each lasting a few minutes, whereas our deployment lasted a month and had dozens of participants. Nevertheless, conceptually, our work used a similar approach to model space in an agnostic manner and captured people’s transition between the various spaces being observed.

### Limitations

The deployment we report took place at a single hospital, lasted 30 days, and did not include all patients and staff at the hospital. We expect that a different hospital or setting might pose different challenges to the study. Still, we also point out that our algorithm analyses do not rely on any particular characteristic or aspect of the hospital itself. We also acknowledge that our data are sparse, meaning that there were many patients and staff that did not have a beacon in our study and were not observed by the system. For this reason, we argue that absolute occupancy rates were not particularly meaningful in our case per se, but we could still compare rooms in terms of how much time our participants spent there, which is a relative measure. Finally, the deployment period was not long enough to capture any potential seasonal effects or any substantial changes to the policies and protocols used at this hospital. A long-term deployment, possibly as part of an A to B study design, would generate more insights on that front.

### Conclusions

In this paper, we described the development, deployment, and evaluation of an indoor localization system for hospitals and clinical settings. We demonstrated how the analysis of longitudinal data could provide operational insights regarding how people inside the hospital move, where they spend their time, and how much do various rooms get occupied. We also discussed how these metrics can be adapted to measure a number of clinical efficiency measures and how they can be used to evaluate changes to policies and protocols in these settings. As part of our future work, we plan to extend our system’s algorithms to evaluate the accumulated *exposure* of clinical staff to infected patients, particularly during the COVID-19 crisis.

## Abbreviations

BLE: Bluetooth low energy
DPU: daily process unity
RFID: radio-frequency identification
RSSI: received signal strength indication
